# Supplementary material for: Long-distance electron transfer in a filamentous Gram-positive bacterium
Source: Nat Commun. 2021 Mar 17;12:1709. doi: 10.1038/s41467-021-21709-z (PMC7969598; doi:10.1038/s41467-021-21709-z)
Supplement: Supplementary file 1 — Supplementary Information [file 41467_2021_21709_MOESM1_ESM.pdf]

Supporting Information

Long-distance electron transfer in a filamentous Gram-positive bacterium

Yonggang Yang[1,2,3], Zegao Wang[4,5], Cuifen Gan[1], Lasse Hyldgaard Klausen[4], Robin Bonné[6], Guannan Kong[1], Dizhou Luo[1], Mathijs Meert[6], Chunjie Zhu[1], Guoping Sun[1], Jun Guo[2], Yuxin Ma[7], Jesper Tataru Bjerg[8], Jean Manca[6], Meiying Xu[1,2,3]*, Lars Peter Nielsen[8], Mingdong Dong[4]*

1 Guangdong Institute of Microbiology, Guangdong Academy of Sciences, 510070, Guangzhou, China

2 State Key Laboratory of Applied Microbiology Southern China, 510070, Guangzhou, China

3 Guangdong Provincial Key Laboratory of Microbial Culture Collection and Application, 510070, Guangzhou, China

4 Interdisciplinary Nanoscience Center (iNANO), Sino-Danish Center for Education and Research (SDC), Aarhus University, Aarhus, Denmark.

5 College of Materials Science and Engineering, Sichuan University, Chengdu 610065, China

6 X-LAB, Hasselt University, BE-3590 Diepenbeek, Belgium

7 School of Life sciences and biopharmaceutics, Guangdong Pharmaceutical University, Guangzhou, 510006, China

8 Center for Electromicrobiology, Aarhus University, DK-8000 Aarhus, Denmark

This PDF file includes:

Supplementary Figure 1 to 8

Other supplementary materials for this manuscript include the following:

Supplementary Data 1

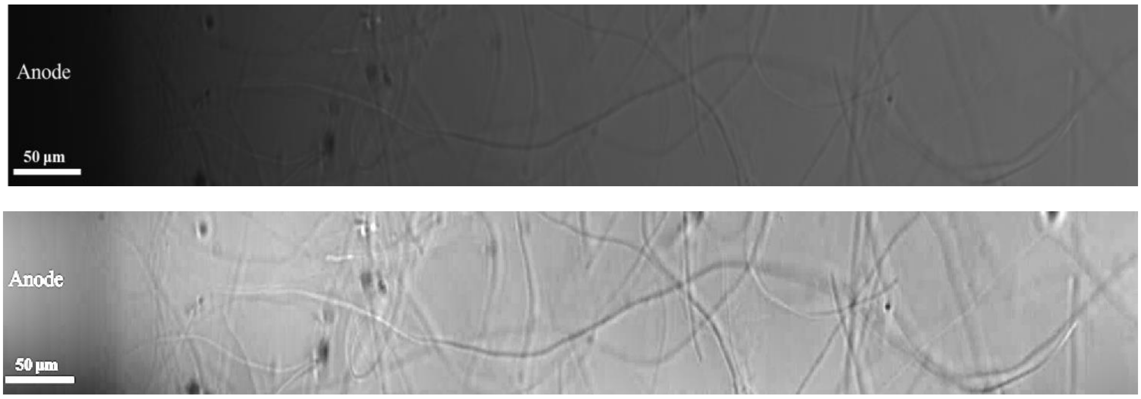

Supplementary Figure 1. Filamentous GY32 cells in a microbial fuel cell. Original (top) and treated with a brightness gradient (bottom).

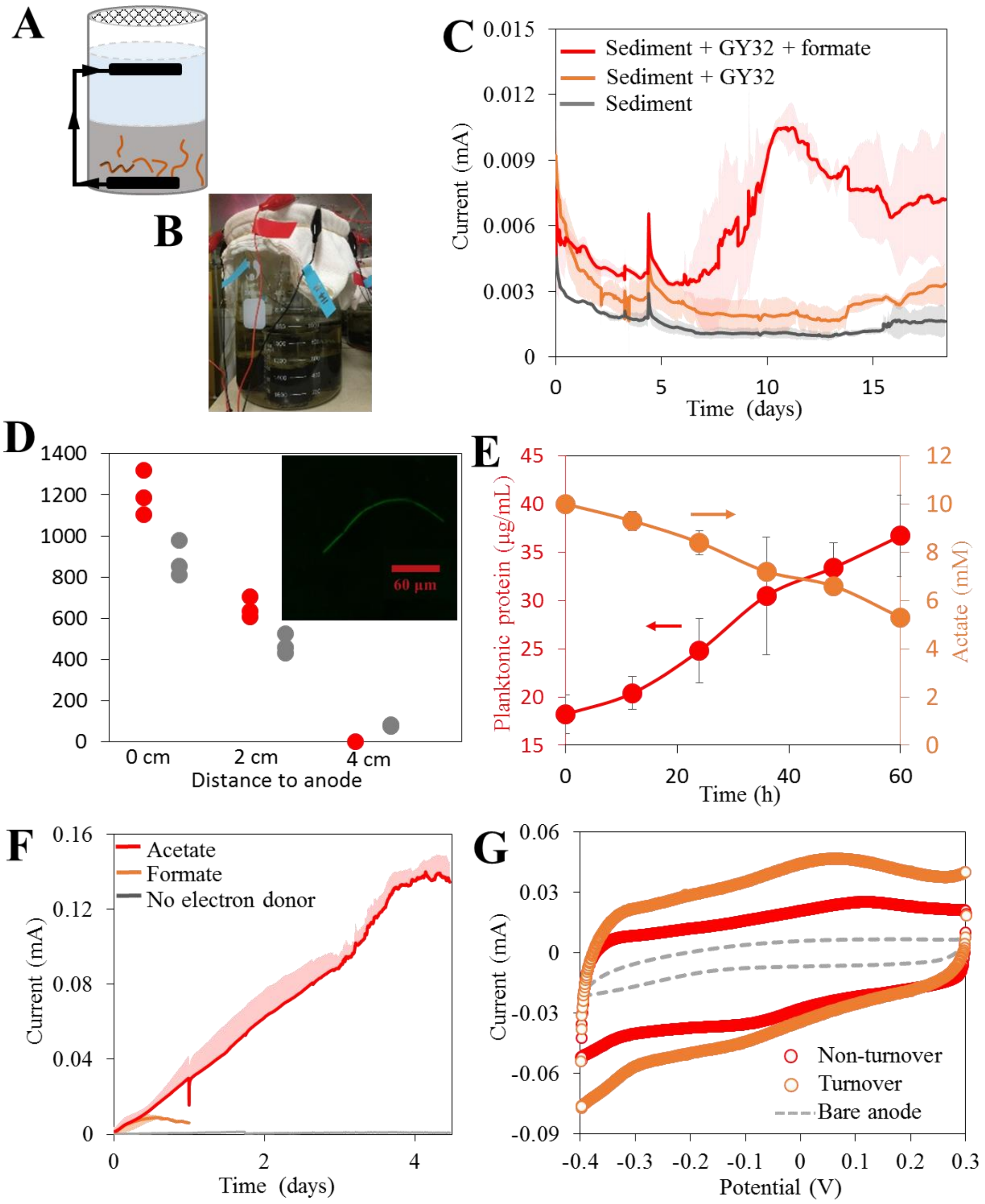

Supplementary Figure 2. Extracellular electron transfer capacity of GY32 in sediment microbial fuel cells. (a) Schematic of a SMFC supplemented with GY32 in the sediments and covered with eight layers of sterilized gauze to avoid the growth of ambient microbes in the sediments. (b) Picture of SMFCs in experiment, triplicate small SMFCs were operated in the same big flask covered by gauze. (c) Current generation by SMFCs containing sterilized sediments with or without GY32. (d) Distribution of GY32 in the sterilized sediments after a 25-day-electricity generation with an average length of approximately 60 µm. The red and grey dots indicate the GY32 number in SMFCs with closed circuit and open circuit, respectively. Insert shows a GY32 cell stained by LIVE/DEAD™ BacLight™ Bacterial Viability Kit (Invitrogen). (e) The biomass (evaluated by cellular protein concentration) increase with acetate consumption in liquid MFCs. The long shape and flocs of GY32 makes it difficult to evaluate the cell growth by optical density or colony counting. Therefore, the cell growth was evaluated by quantifying the protein of the planktonic culture with a Bradford protein assay kit. (f) Current generation by strain GY32 using a polarized anode (0.4 V vs standard

hydrogen electrode) as electron acceptor and acetate or formate as electron donor. (g) Representative cyclic voltammetry curves of anodic GY32 biofilms under turnover and non-turnover conditions. c-f shows the results from 3 biological replicates (n=3) as mean ± SD. Source data are provided as a Source Data file.

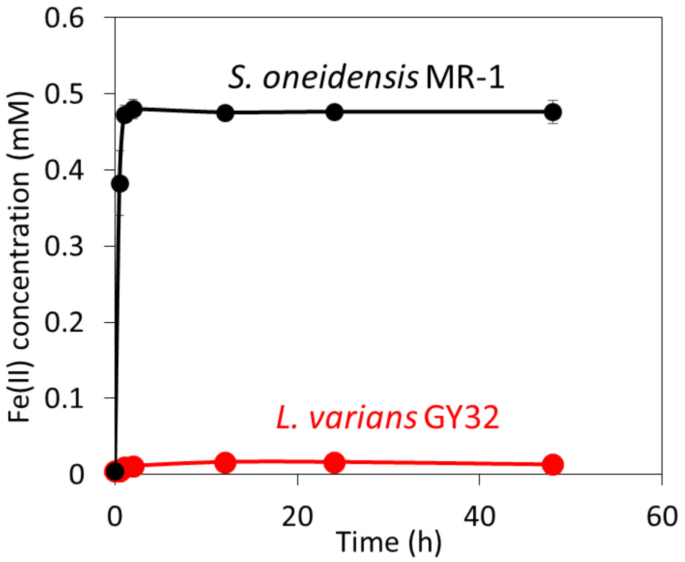

Supplementary Figure 3. H_2 reduction by *L. varians* GY32 *Shewanella oneidensis* MR-1. *L. varians* GY32 and *Shewanella oneidensis* MR-1 were inoculated in sterilized PBS buffer (pH 7.0, initial OD_{600} = 0.1) containing 1 mM of ferric citrate in serum bottles. The buffer was firstly bubbled with nitrogen to remove oxygen (verified by O_2 microelectrode, H2-25-7343, Unisense) and then bubbled with pure hydrogen until the H_2 concentration reached 0.5 mM (measured by H_2 microelectrode, H_2-25-7343, Unisense). The serum bottles were sealed with butyl rubber and aluminium caps. *S. oneidensis* MR-1 was used as a positive control as it has the capability to use H_2 as electron donor to reduce ferric citrate (Liu et al., 2002, Biotechnol Bioeng 80: 637–648). n = 3. Source data are provided as a Source Data file.

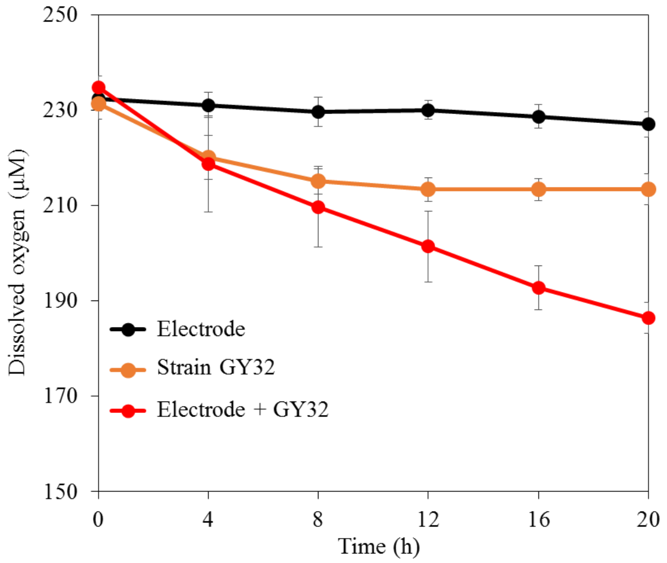

Supplementary Figure 4. O_2 reduction profile of *L. varians* GY32 using electrode polarized at 0.1 V (vs standard hydrogen electrode) as electron donor. In the BCES with polarized electrode and GY32, the oxygen reducing rate (2.5 ± 0.3 μM/h) was much higher than the BCES with only GY32 (0.9 ± 0.2 μM/h) or polarized electrodes (0.3 ± 0.1 μM/h) (n = 3 independent bacterial culture, plot shows mean ± SD). The oxygen reduction by PBS-washed GY32 without a polarized electrode may be attributed to the inherent nutrients stored within the cells. Source data are provided as a Source Data file.

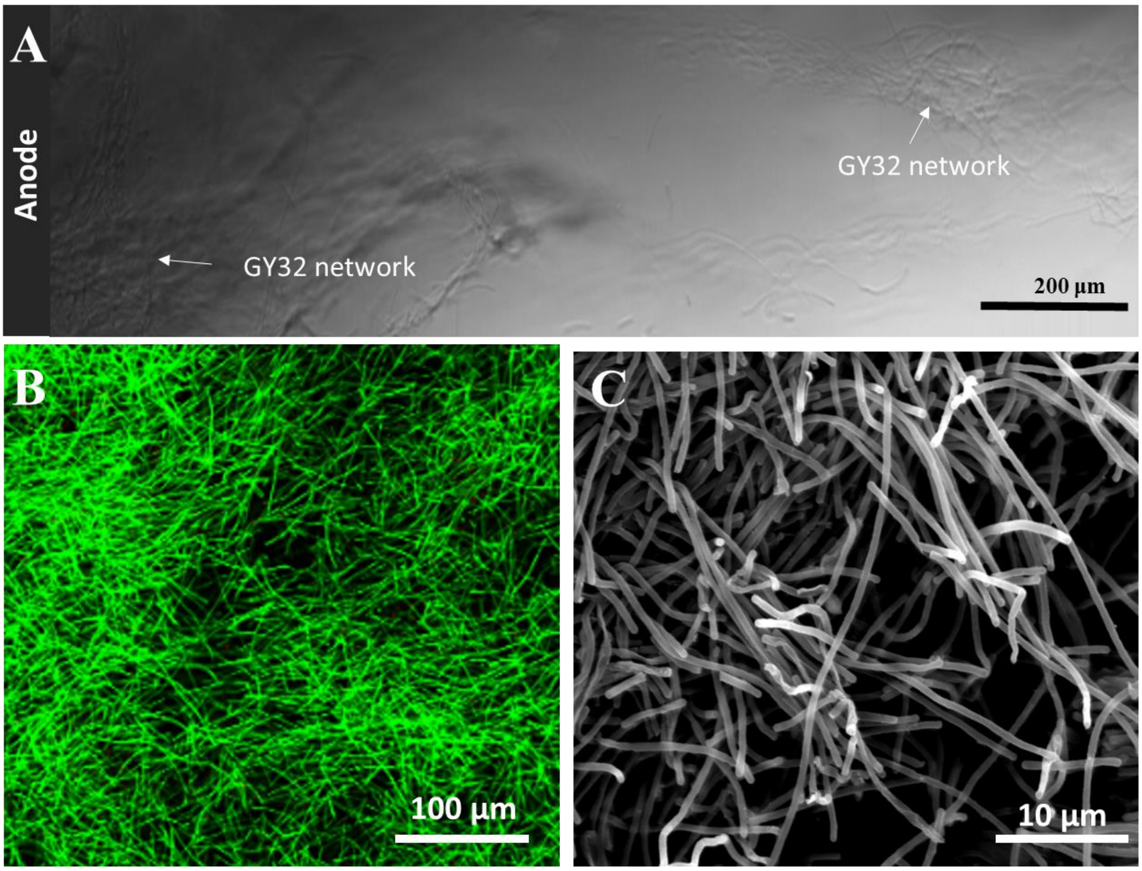

Supplementary Figure 5. Filamentous structure of GY32 cellular networks at anode surface. (A) GY32 cellular networks around the anode surface in MFCs containing liquid culture. (B) GY32 biofilms grown on the anode (stained by LIVE/DEAD™ BacLight™ Bacterial Viability Kit, Invitrogen). (C) Anode GY32 biofilms observed under scanning electronic microscope.

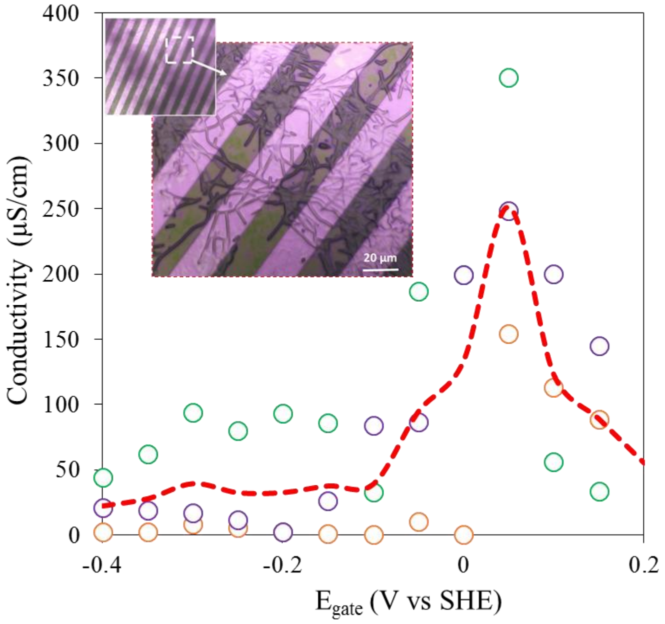

Supplementary Figure 6. Conductivity of GY32 biofilms based on electrochemical gating measurements. The doted curves indicate conductivities of three biofilm samples and the dashed line indicates the averaged conductivities of the three samples (E_{gate}: gate potential). Insert shows the GY32 biofilms on IMAs. The image with a white square shows a zoom-out view of the biofilms in which the dark-purple areas indicate dense biofilms and light-purple areas indicate rare biofilms. The white arrow indicates a zoom-in of the white square and the dark-purple filaments indicate GY32 cells. Source data are provided as a Source Data file.

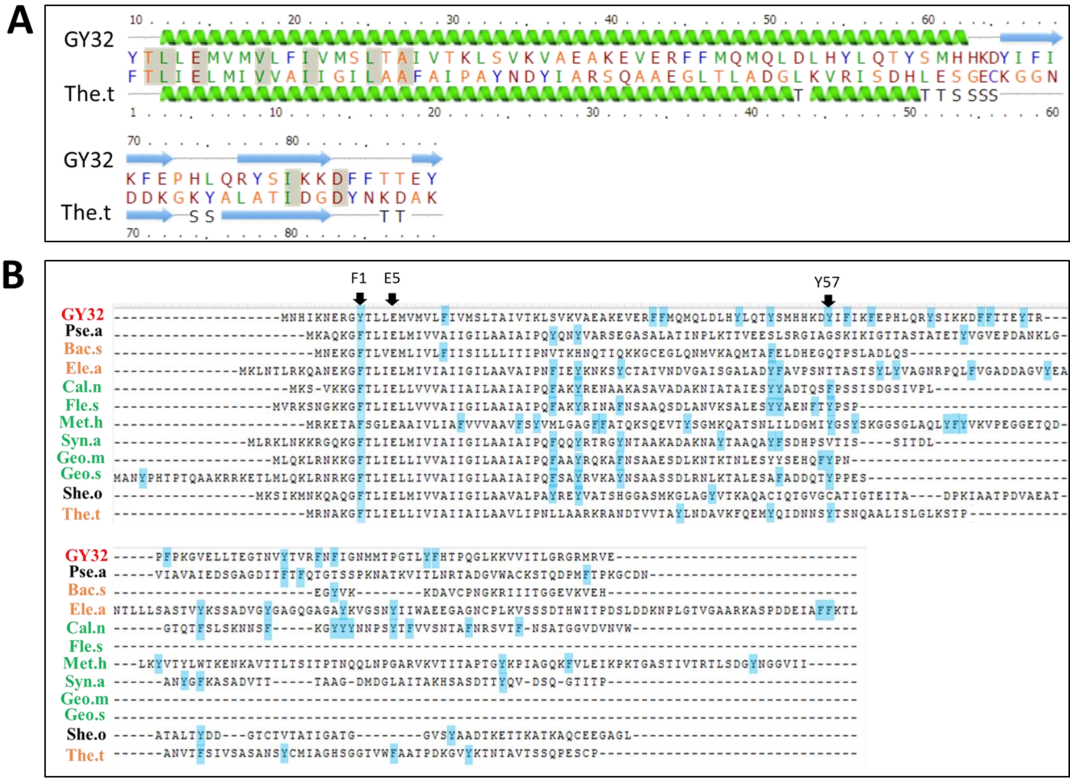

Supplementary Figure 7. GY32-ComGD structure. (A) Predicted secondary structure of GY32-ComGD (protein id: WP_024361267.1, https://www.ncbi.nlm.nih.gov/protein/636839507) using Phyre2 (http://www.sbg.bio.ic.ac.uk). The template structure is type IV pilin of *Thermus thermophiles* (The.t, https://www.ncbi.nlm.nih.gov/protein/AAM55486) with a confidence of 99.3% and a coverage of 82%. (B) Aromatic amino acids (marked in blue) in GY32-ComGD and pilins from several other microorganisms. The position of F1, E5 and Y57 are indicated by arrows. The proteins used here is: GY32 (WP_024361267.1 of *Lysinibacillus varians* GY32), Pse.a (P04739 of *Pseudomonas aeruginosa*, https://www.ncbi.nlm.nih.gov/protein/P04739), Bac.s (B657_24710 of *Bacillus subtilis*, https://www.ncbi.nlm.nih.gov/protein/402481913), Ele.a (Ga0183576_10762 of Ca. *Electronema* sp. GS, https://www.ncbi.nlm.nih.gov/protein/1578860704), Cal.n (Calni_0149 of *Calditerrivibrio nitroreducens*, https://www.ncbi.nlm.nih.gov/protein/312938870), Fle.s (Flexsi_2288 of *Flexistipes sinusarabici*, https://www.ncbi.nlm.nih.gov/protein/336108085), Met.h (Mhun_3140 of *Methanospirillum hungatei*, https://www.ncbi.nlm.nih.gov/protein/88189828), Syn.a (SYN_00814 of *Syntrophus aciditrophicus*, https://www.ncbi.nlm.nih.gov/protein/85723214), Geo.m (Gmet_1399 of *Geobacter metallireducens*, https://www.ncbi.nlm.nih.gov/protein/78193866), Geo.s (GSU1496 of *Geobacter sulfurreducens*, https://www.ncbi.nlm.nih.gov/protein/AAR34870.1), She.o (WP_011070777.1 of *Shewanella oneidensis*, https://www.ncbi.nlm.nih.gov/protein/WP_011070777.1?report=genpept), The.t (AAM55486 of *Thermus thermophiles*, https://www.ncbi.nlm.nih.gov/protein/AAM55486). Green names indicate verified conductivity, black names indicate no conductivity, yellow names indicate unknown conductivity.

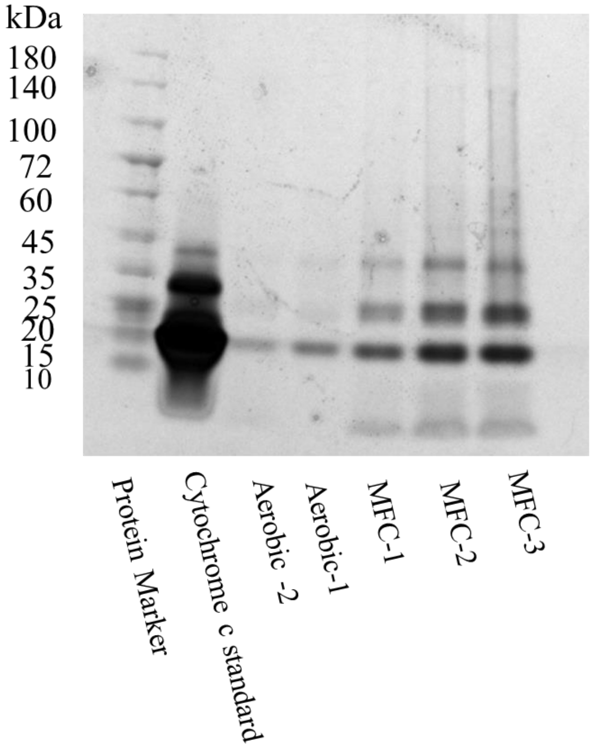

Supplementary Figure 8. Polyacrylamide gel electrophoretic separation of the extracellular proteins stained by cytochrome-specific 3,3',5,5'-tetramethylbenzidine. Equal protein amount (100 μg) was loaded in each lane of a 12% SDS-PAGE gel. It can be seen that GY32 grown in MFCs generated higher *c*-type cytochrome concentration than GY32 grown under aerobic condition. Lane MFC-1, MFC-2, MFC-3 show samples from three MFCs, Lane aerobic-1, aerobic-2 show samples from aerobic culture of GY32.
