# Supplementary material for: Long-distance electron transfer in a filamentous Gram-positive bacterium
Source: Nat Commun. 2021 Mar 17;12:1709. doi: 10.1038/s41467-021-21709-z (PMC7969598; doi:10.1038/s41467-021-21709-z)
Supplement: Supplementary file 3 — Description of Additional Supplementary Files [file 41467_2021_21709_MOESM3_ESM.pdf]

Description of Additional Supplementary Files

File Name: Supplementary Data 1

Description: Transcriptomic comparison of Lysinibacillus varians GY32 respiring with oxygen and MFC-anode.
